# Three categories of similarities between the placenta and cancer that can aid cancer treatment: Cells, the microenvironment, and metabolites

**DOI:** 10.3389/fonc.2022.977618

**Published:** 2022-08-18

**Authors:** Huiyuan Pang, Di Lei, Yuping Guo, Ying Yu, Tingting Liu, Yujie Liu, Tingting Chen, Cuifang Fan

**Affiliations:** ^1^ Department of Obstetrics and Gynecology, Renmin Hospital of Wuhan University, Wuhan, China; ^2^ Department of Obstetrics and Gynecology, First Affiliated Hospital of Gannan Medical University, Ganzhou, China

**Keywords:** Decidua, placenta, TME (tumor microenvironment), cancer, cachexia, placenta diseases

## Abstract

Cancer is one of the most harmful diseases, while pregnancy is a common condition of females. Placenta is the most important organ for fetal growth, which has not been fully understand. It’s well known that placenta and solid tumor have some similar biological behaviors. What’s more, decidua, the microenvironment of placenta, and metabolism all undergo adaptive shift for healthy pregnancy. Interestingly, decidua and the tumor microenvironment (TME); metabolism changes during pregnancy and cancer cachexia all have underlying links. However, whether the close link between pregnancy and cancer can bring some new ideas to treat cancer is still unclear. So, in this review we note that pregnancy may offer clues to treat cancer related to three categories: from cell perspective, through the shared development process of the placenta and cancer; from microenvironment perspective, though the shared features of the decidua and TME; and from metabolism perspective, through shared metabolites changes during pregnancy and cancer cachexia. Firstly, comparing gene mutations of both placenta and cancer, which is the underlying mechanism of many similar biological behaviors, helps us understand the origin of cancer and find the key factors to restore tumorigenesis. Secondly, exploring how decidua affect placenta development and similarities of decidua and TME is helpful to reshape TME, then to inhibit cancer. Thirdly, we also illustrate the possibility that the altered metabolites during pregnancy may reverse cancer cachexia. So, some key molecules changed in circulation of pregnancy may help relieve cachexia and make survival with cancer realized.

## Introduction

Cancer, similar to many other diseases, can be divided into stages of primary lesions and metabolism changes throughout the whole body. The earlier process of tumors is tumorigenesis, which leads to the development of local cancer lesions; the later stages of almost 50-80% of cancer cases involve cachexia, which means wasting conditions with skeleton loss, adipose tissue loss, muscle atrophy and appetite loss (1). Cachexia is most mortal metabolism shift of cancer, responsible for 20% of tumor-related deaths (1), so we mainly discuss cachexia when came to cancer metabolism shift. Though pregnancy is a physiological state, it can also be thought of a two-stage process: the first stage involving synergistic development of the placenta and decidua, and the later-stage involving an adaptive metabolism shift.

Some similarities between the placenta and tumors have been reported (2–4) and are thus only briefly discussed in our review. As early as 2004, the development of the placenta was suggested to be akin to ‘pseudo-tumorigenesis’ (5). Both the placenta and tumors bear a high level of genetic mutations (6, 7) and feature epithelial-mesenchymal transition (EMT), invasion and cell–cell fusion (8); both can form their own blood vessels through vascular mimicry (VM) and angiogenesis to meet their massive energy consumption demands (9, 10). Even with so many similarities, the placenta is under precise regulation and can be rapidly separated from the decidua during labor, while the development of cancer cells is chaotic, uncontrolled and harmful.

The second category of similarities is between the decidua and TME. The decidua is the environment regulating trophoblast growth and differentiation, and the TME provides a similar environment for cancer cells. Trophoblasts and cancer cells both rely on the environment to provide nutrients, and they can crosstalk with and sculp their environment (11). The decidua and TME both contain complex mixtures of cells (fibroblasts and immune cells), extracellular matrix (EMC), and cytokines produced by these cells and feature unique stressors, such as hypoxia (12, 13).

Discrepancies between the placenta and decidua are the root of placental diseases, which are caused by abnormal implantation depth. Shadow implantation and insufficient decidualization can be observed in recurrent spontaneous abortion (RSA) and preeclampsia (PE) (14), while thinner decidua and over invasion placenta can be seen in abnormally invasive placenta (AIP). Given the features shared between the placenta and cancer and between the tumor microenvironment (TME) and decidua, we suggest that cancers and placental diseases can be categorized into similar disease stages. Tumors exist on a spectrum, which includes a variety of placental diseases. This opinion is better for future research to explore tumorigenesis. What’s more, the regulation role of decidua needs more attention, since decidua can both promote placenta development and restrict over invasion.

The third category of similarities is related to the shared reprogramming of metabolism that occurs during pregnancy and cancer cachexia, which involves many of the same molecules, signaling pathways. Embryos are similar to tumors in that they both have massive energy requirement, but the results for each are different. These patterns, provides a new idea for tumor treatment: can the metabolites changed that occur during pregnancy have antitumor effects?

Researches on cancer can fall into the following categories: solid tumor, TME, and cancer cachexia. Similarly, pregnancy research can fall into the following categories: placenta, decidua, and metabolism reprogramming during pregnancy. These three categories are shared between pregnancy and cancer, and research investigating these similarities may increase our understanding of placental diseases and cancer. So, there are three key factors in pregnancy that can be exploited for cancer treatment.

## Cell

Trophoblasts and cancer cells can undergo epithelial-mesenchymal transition (EMT) and invasion, cell–cell fusion, and immune escape. In cancer, EMT is an essential step for invasion and metastasis and has been researched for many years (15). In placental development, cytotrophoblasts (CTBs) can undergo pseudo-EMT and convert into extravillous trophoblasts (EVTs) (16–18). EVTs are invasive and remodel the uterine spiral artery. CTBs can also form syncytiotrophoblasts (STs) by cell–cell fusion, which occurs in cancer (19, 20) to respond to gene damage after chemotherapy or radiation (21). Cell–cell fusion in the placenta may help STBs defend against oxidative stress-induced damage caused by blood flow from the uterine spiral artery. Half of the genes of trophoblasts come from the father and can be seen as foreign, similar to cancer cells. Both express special surface antigens to escape attack by immune cells (22).

However, why these cells share so many similarities is still unknown. Exploring the specific factors driving these similarities is important. Recent researches on gene mutations in the placenta has have revealed the underlying mechanisms of tumor-like behaviors. In 2017, Alexander Meissner’s team analyzed changes in global remethylation profiles from mouse preimplantation embryos to early epiblasts and extraembryonic ectoderm. The researchers found that placental development shares some signaling pathways with cancer (23). Different samples of the same placenta exhibited genetically distinct clonal expansion (24). More interestingly, genetic alterations seen in placenta can be observed in childhood cancers, especially neuroblastoma and rhabdomyosarcoma (25). In 2021, Professor Tim HH Coorens published an article in Nature pointing out that extensive mutagenesis in placental tissues and mosaicism are typical features of placental development (6, 24). The placenta can tolerate gene mutation without causing maternal harm, while cancer cells also tolerate chromosomal aberrations but are harmful. An understanding of the mechanism underlying how trophoblasts tolerate chromosomal aberrations will provide new ways to kill cancer. The cells forming the placenta and embryo was separated within the first several weeks of human development (26), and mutations in the placenta are not present in the fetus (27); as such, the placenta has been suggested as a so-called dumping ground for cells with genetic defects located in the decidua, which can then be removed during delivery. These concepts may enlighten the understanding of cancer—which could be seen as a state in which such defective cells are present throughout the body instead of limited to one area. They cannot be put in a garbage like placenta or decidua. Whether we can reshape the microenvironment of cancer and sequester these defective cells will be reviewed in the following section.

## Microenvironment

### Similar functions of the decidua and TME

The formation of the maternal-fetal interface depends on coordinated maternal and neonatal development, that is, the coordinated development of the decidua and placenta. From the 15th to 19th day of the menstrual cycle, called the secretory phase, endometrial stromal cells proliferate and differentiate into large, round, cytoplasmic, multinucleated decidual cells, which participate in decidualization (28); this process increases the ease of placental implantation.

The microenvironment, decidua and TME share many similar functions. Their important roles in placental and cancer development have drawn much attention (29). The decidua and TME share multiple regulatory processes, including modulation of epigenetics and regulation of EMT, invasion, immune tolerance, and nutritional supply.

#### Epigenetic regulation

A study showed that cell column trophoblasts (CCTs) of patients with preeclampsia have transcriptomic differences compared with those of normal pregnant women, but when the two groups of CCTs were separated from the original decidual environment and cultured in vitro, the transcriptomic differences between the two groups gradually disappeared (30). It has been demonstrated that the decidua, as the microenvironment for embryo implantation, significantly affects the epigenetics of trophoblasts (31, 32). The earliest interactions between the blastocyst and decidua initiate gene expression reprogramming of the trophectoderm. Mouse blastocysts cocultured with the human endometrial cell line Ishikawa have altered expression of Cdx2 and Gata3 (33), which are critical for cell differentiation. Although no study has dynamically assessed the regulation of the trophoblast transcriptome by the decidua during the entire gestational period, it is certain that the decidua can significantly regulate the transcriptome of trophoblasts.

Epigenetic alterations are also present in tumors in addition to genetic mutations (34, 35). Cancer can reshape the TME, and the TME can affect the cancer cell epigenetics in turn. Pancreatic cancer TME factors promote cancer stemness via the SPP1-CD44 axis (36). In gastric cancer, TME infiltration is closely associated with cancer cell m-6-A regulator-mediated methylation (37).

Translational control of mRNAs allows tumor cells to dynamically adapt the TME in response to anticancer therapies (38). An understanding of the decidua may enable modification of the mRNA translation of tumors to force them to shift from malignant to benign.

#### Immune tolerance

The status of EVTs, the outermost structure of the embryo that interfaces with the maternal decidua, is important for successful pregnancy. During the differentiation from CTBs to EVTs, HLA-G molecule expression gradually increases. HLA-G belongs to the human leukocyte antigen family, is specifically expressed at the maternal-fetal interface, and mediates the immune tolerance of the placenta. HLA-G is only expressed by EVTs and interacts with the decidual natural killer (dNK) cell inhibitory receptor killer immunoglobin-like receptor (KIR) KIR2DL4 (39) and LILRB (40) to protect trophoblast cells from NK-mediated cytolysis. dNK cells can express KIRs that can interact with HLA-C expressed by trophoblast cells to maintain immune balance at the maternal-fetal interface.

Both trophoblast and tumor cells are able to escape attack by the immune system. Tumor cells have a variety of harmful gene mutations, and their immune escape mechanisms are complicated. HLA-G can also bind to the NK inhibitory receptor KIR2DL4 in breast cancer (41). Tumor cells can not only avoid being attacked by escaping immune system notice but also secrete TGF-β or other immunosuppressive factors to inhibit the actions of infiltrating cytotoxic T lymphocytes (CTLs) and NK cells (42) and recruit inflammatory cells with aggressive immunosuppressive effects, including regulatory T cells (Tregs) (43) and myeloid-derived suppressor cells (MDSCs). Both inhibit the action of cytotoxic lymphocytes (44).

#### Nutrient supply

**Figure 1:**
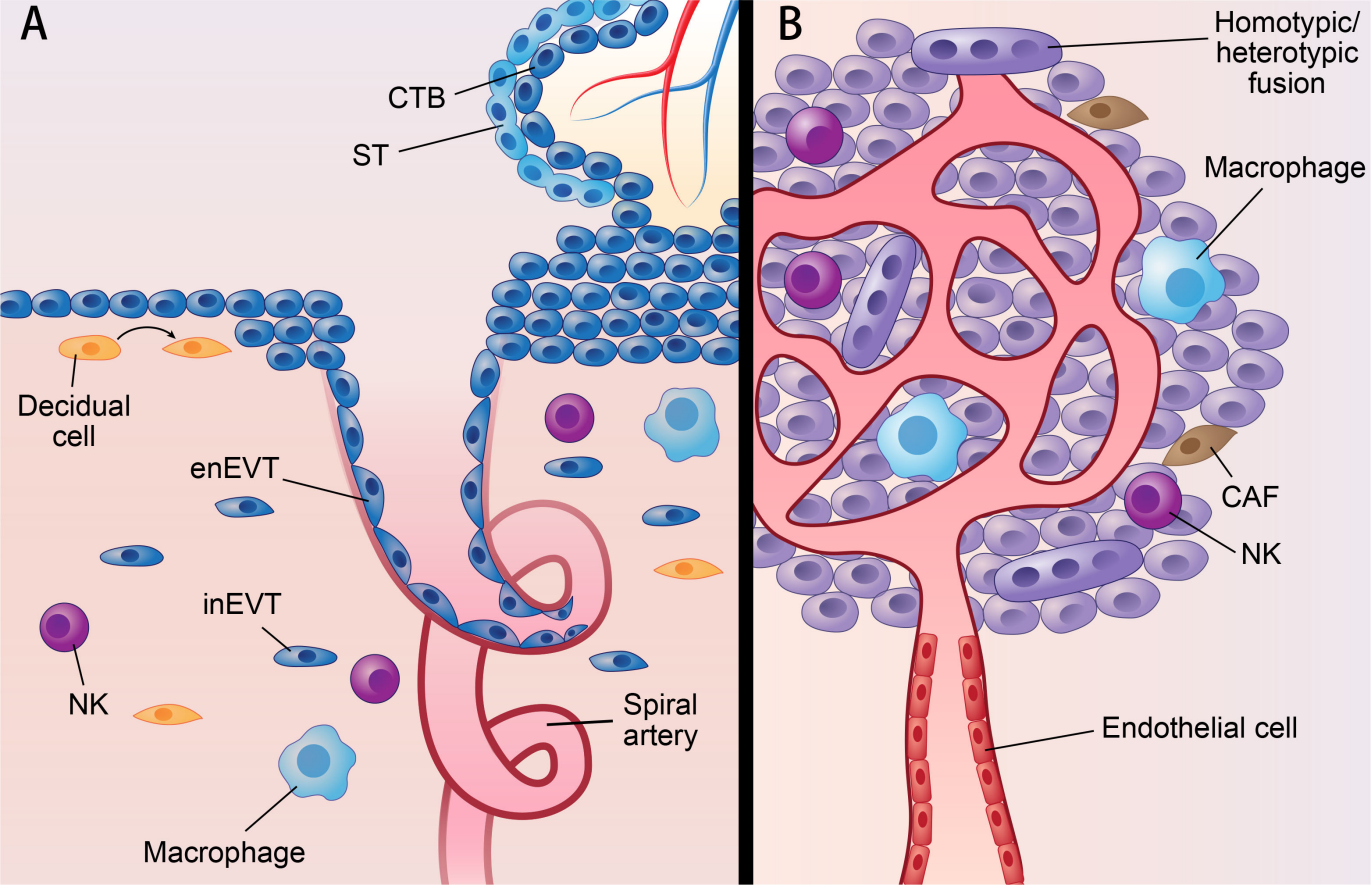
Parallels between placenta and cancer; decidua and TME. Parallels between placenta and cancer; decidua and TME: **(A)** represents villous of placenta and decidua. **(B)** represents solid cancer and TME. Figure 1 shows similarities of placenta and cancer; decidua and TME. In placenta, ST originates from CTB by cell-cell fusion, while cancer can fuse with other cells by homotypic/heterotypic fusion. CTB develops into EVT via partial EMT which also occurs in cancer. Decidua and TME also share many components, such as NK cell; macrophage; fibroblast (decidual cell in decidua; CAF in TME). What’s more, spiral artery remodeling in decidua shared features with VM, since both of them mean channels without vascular endothelial cell. CTB, cytotrophoblast; ST, syncytiotrophoblast; EVT, extravillous trophoblast; enEVT, endovascular EVT; inEVT, interstitial EVT; CAF, cancer-associated fibroblasts; NK, natural killer cell; VM, vascular mimicry.

Before vascular formation, trophoblasts and tumor cells exist in their microenvironment and gain nutrients directly from the surrounding tissue (45, 46). After vascular formation, both trophoblasts and tumor cells induce VM. VM is a newly found process by which tumor cells generate additional blood supply that is different from traditional angiogenesis (47); with VM, the tumor blood supply does not depend on invasion of the vascular endothelium, and the tumor itself forms channels (48). VM indicates the formation of tumor cell-lined vasculature, which is different from endothelium-lined vasculature (10). Tumor cell-lined vasculature mainly has four features: expression of endothelium biomarkers, such as VE-cad (48); formation of an extracellular matrix (ECM)- rich network; presence of erythrocytes and plasma; ability to undertake material exchange (10). VM partly explains the poor efficacy of and resistance to angiogenesis inhibitors in the treatment of tumors (49–51). Trophoblasts also have similar physiological characteristics to tumor cells. The trophoblasts that remodel the uterine spiral artery gradually express the VM marker VE-cad. Therefore, the vessels that form in the placenta are also thought to be formed by a kind of VM (9, 52, 53) instead of traditional endothelium-lined vessels (**Figure 1**).

Pericytes surround blood vessels. They have finger-like protrusions that surrounding the vascular endothelium, providing support for blood vessels and controlling vasculature stability and permeability (54). Tumor blood vessels are mainly composed of vascular endothelial cells and pericytes, and pericytes play a key role in angiogenesis and vessel maturation and have been noted as potential targets for tumor therapy (55–58). In the placenta, pericytes preferentially cover endothelial cell–cell junctions. They tend to be most common in regions furthest away from the trophoblast and less common in regions adjacent to the trophoblast, since most transfer occurs here (59). Ang/Tie is an important pathway for signal communication between pericytes and endothelial cells (58), and the vascular endothelium -secreted growth factor PDGFb recruits PDGFRb-expressing pericytes (60). PDGF and its receptors play an important role in tumor pericyte recruitment and communication. Inhibitors of PDGF signaling can disrupt vascular integrity and function by affecting pericytes.

### Similar components of the decidua and TME

The decidua contains decidual stromal cells (DSCs) and other immune cells, 70% of which are dNK cells, as well as many kinds of noncellular components, such as cytokines, ECM components and metabolites (**Figure 1A**). Moreover, the decidua also contains unique features, such as a concentration gradient of oxygen (61) and specific levels of cytokines. It is well known that, similar to the decidua, the TME also contains cells and noncell components. The cells include cancer-associated fibroblasts (CAFs) and immune cells; the noncell components include ECM, cell-secreted products and metabolites (**Figure 1B**). The TME can also contains unique environmental stressors, such as hypoxia.

#### Fibroblast and fibroblast-secreted cytokines

DSCs and CAFs play important roles in decidualization and TME modulation (62). Both of these cells and their target cells can reshape the other. A balance between these cell types is necessary for placental development, decidualization, tumorigenesis and TME modulation. The differentiation of DSCs and CAFs involves cell transformation. DSCs experience mesenchymal-epithelial transition (MET) (63), and CAFs experience macrophage-myofibroblast transition (MMT) (60). MET of DSCs occurs spontaneously in humans and is regulated by sex hormones, while the transformation of CAFs requires triggering by cancer cells. Whether we can prevent CAF formation and inhibit cancer remains to be determined.

The cytokines they secrete perform fundamental roles in cell-to-cell interactions. The C-X-C motif chemokine ligand (CXCL) family and C-X-C chemokine receptor (CXCR) family are important in this crosstalk. CXCLs are secreted by trophoblast cells and tumor cells, as well as cells in the decidua and TME (64–66). CXCLs/CXCRs play important roles in the interactions at the maternal-fetal interface (67) and cancer-TME interface. In the decidua, CXCL12 interacts with CXCR4 to promote invasion and suppress apoptosis of trophoblasts (68), while CXCL14, another member of the CXCL family, can block these effects. Aberrant CXCL/CXCR expression has also been observed in placental diseases. CXCL12 and CXCR4/CXCR7 are upregulated in AIP, and their expression can be detected in circulation (69). Snail promotes ovarian cancer progression by recruiting MDSCs via CXCR2 ligand upregulation (70). CCL2, expressed by DSCs, macrophages and EVTs, can help recruit T cells to the maternal-fetal interface (71, 72) and recruit MDSCs to the TME (73), which can be observed in lung cancer and pancreatic ductal adenocarcinoma (74–77). Many signaling pathways are shared between these processes, such as the Wnt (78–83), Notch (84–88), and Hippo signaling pathways (81, 84, 89–91).

#### Noncellular components

ECM stiffening and degradation drive cancers (92), while decidualization is associated with extensive ECM remodeling (93). ECM remodeling heavily relies on matrix metalloproteinases (MMPs). MMPs can be secreted by trophoblast, decidual, and cancer cells and cell in the TME (94), playing a pivotal role in collagen and fibronectin degeneration (95, 96). Recent studies have indicated that growth factors are also involved in interactions with ECM proteins and proteolytic enzymes, for example, the IGF–IGFR–IGFBP axis (97). ECM remodeling is important for cell invasion and immune cell recruitment.

Hypoxia is an important characteristic of the decidua and TME. In the first few weeks of placental development, CTBs form trophoblast plugs that block the uterine spiral artery, disturbing blood flow into the intervillous space and resulting in a limited oxygenation environment around trophoblast cells (98). A moderately hypoxic environment is believed to promote CTB exit from the cell cycle, induction of EMT, and acquisition of invasive ability.

Hypoxia is also very important for tumors (99, 100). Hypoxia can induce many tumor processes, such as invasion, vasculogenesis, angiogenesis and VM, to generate a nutrient supply system (101). Hypoxia can increase the expression of the hypoxia-inducible factors (Hifs), which is associated with a poor prognosis (102–104). Hifs are a key link between hypoxia and different cell behaviors and are important factors promoting trophoblast invasion.

Importantly, these factors also influence each other (105). Hypoxia promotes high CXCR expression, which in turn promotes invasion (106). Moreover, hypoxia may contribute to VM (51, 107). The ECM provides an environment that allows T-cell invasion into tissues or directly inhibits T-cell proliferation to support and suppress adaptive immune responses, respectively (108).

### Cancer and placental diseases

The decidua can regulate and support the growth and differentiation of trophoblast cells. Discrepancies between the placenta and decidua can cause placental diseases, such as RSA and PE (14).

**Figure 2:**
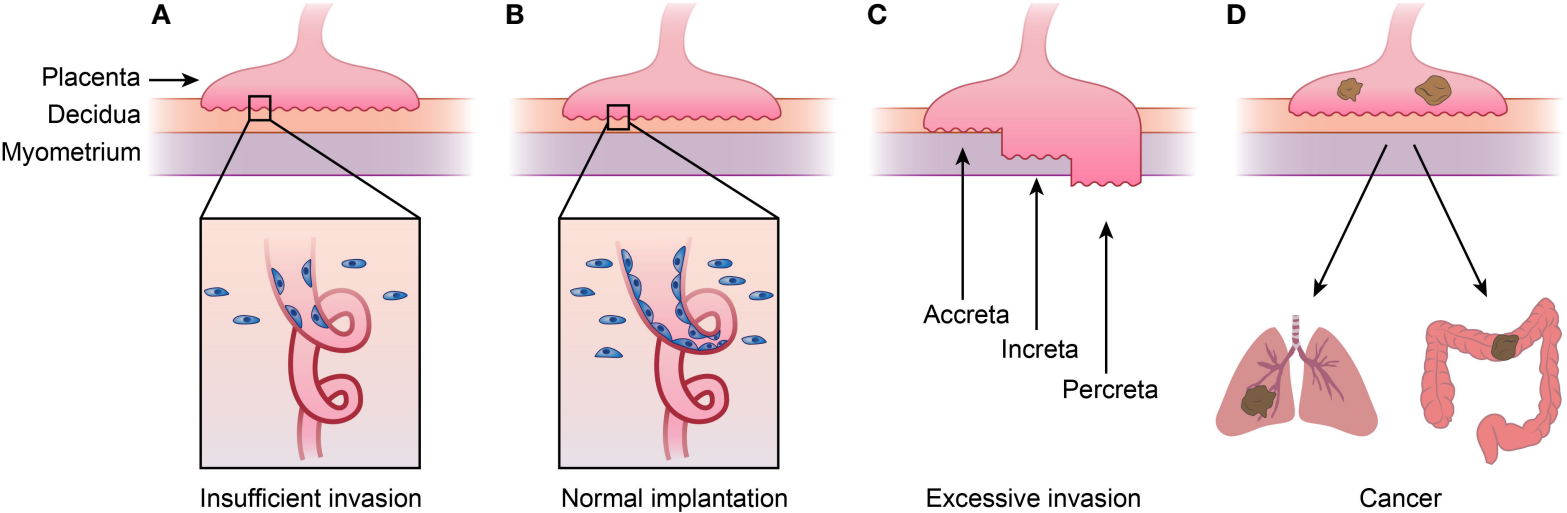
Placenta diseases and Choriocarcinoma. **(A)**shows placenta diseases caused by insufficient invasion of trophoblast and spiral artery remodeling, which is caused by both unhealthy trophoblast and decidua. **(B)** represents normal implantation, and **(C)** shows AIP caused by excessive invasion of trophoblast and thinner decidua. AIP can be categorized into placenta accreta, placenta increta and placenta percreta depending the depth of implantation. **(D)** shows choriocarcinoma, meaning cancer happened of trophoblast which tend to metastasis to lung or other organs. This figure shows that placenta diseases and choriocarcinoma or even other kind cancers are in a spectrum. AIP, abnormally invasive placenta.

RSA, defined as two or more consecutive pregnancy losses before 20 weeks of gestation, occurs in 2-5% of women during their reproductive years (109, 110). An impaired decidua can lead to RSA (111, 112). PE is another common obstetric complication that occurs in 2%-8% of pregnant women (113). Patients with two miscarriages are more likely to have preeclampsia (114). PE occurs when in the second half of pregnancy, the systolic blood pressure (SBP) changes to >=140 mmHg and/or the diastolic blood pressure (DBP) changes to >=90 mmHg in patients whose previous blood pressure was normal (115). During placental development, trophoblast cells invade the uterine spiral artery and replace vascular endothelial cells, expanding the diameter of the uterine spiral artery and reducing vascular resistance to meet the nutrient needs of the fetus (116). The preclinical stages of RSA and PE both involve defective trophoblast invasion and insufficient decidualization (31, 117–119). Failure of implantation causes fetal loss; insufficient uterine spiral artery remodeling can cause trophoblast ischemia, and the injured placenta releases inflammatory factors and/or exosomes, causing hypertension (120) (**Figures 2A, B**). Although it is regrettable that impaired decidualization and insufficient invasion of trophoblast cells lead to poor pregnancy outcomes, the defects in gene expression of the injured decidua and placenta revealed by transcriptional profiling may reveal factors that can be targeted to treat cancer. Whether the factors in the decidua of RSA and PE can have antitumor effects is an interesting point to research in the future.

In another placental disease, AIP, the decidua thins and the placenta invades the deeper uterine wall (121), which are processes similar to those seen in malignant tumors (122). AIP is now recognized as a series of diseases. According to the depth of implantation, AIP is divided into three types: placenta accreta (PA; attachment to myometrium without intervening decidua), placenta increta (PI; invasion into the myometrium), and placenta percreta (PP; invasion through the uterine wall). AIP affects 1 in 1000 pregnant women (123) (**Figures 2B, C**). The occurrence of AIP is closely related to the absence of decidua caused by cesarean delivery (124–127). Uterine scarring after c-section can lead to endometrial defects and failure of normal decidualization in the incision area, which leads to an abnormal depth of implantation (128).

As mentioned above, the decidua not only promotes or inhibits the invasion of trophoblasts but also ensures that process occur within a suitable depth. The decidua can not only provide the proper environment for trophoblasts to invade but also inhibit villi from invading beyond 2/3 of the uterine wall. During delivery, the placenta is rapidly released from the decidua, acting as a container that temporarily holds and provides energy for the embryo while restricting overgrowth. However, there is no equivalent structure to the decidua in tumors. The diseases above are in a spectrum of diseases. The absence of the decidua causes placental overgrowth, and decidual dysplasia restricts the development of the placenta. These ideas imply that the placental microenvironment in PE or RSA may inhibit tumor growth (**Figure 2**).

## Metabolites

Placenta and cancer both feature metabolism reprogramming throughout the body. During pregnancy, in addition to changes in the female reproductive system, many systems change to meet the increased body weight and energy requirements of the fetus (129). The basal metabolic rate of the mother increases significantly, and increased blood volume, hyperglycemia, and a positive nitrogen balance also occur. Similarly, tumors not only affect the primary and metastatic tumor sites but also result in changes in the whole body. Cachexia mainly manifests as abnormal metabolism, especially the loss of skeletal muscle (130, 131). Removing tumors or effectively treating them can partially ameliorate cancer cachexia (132–134), suggesting that cancer cachexia is caused by tumor cell-released factors. 50%-80% patients with advanced tumors will suffer cachexia (1), which can also be seen in a variety of chronic diseases, such as chronic heart failure. Importantly, this depleted state cannot be reversed by the additional intake of more nutrients. Therefore, there is a shared mechanism underlying cancer and other disorders. From the perspective of energy metabolism, tumors and other wasting-related metabolic diseases exist on the same disease spectrum. Cachexia involves many of the same adaptive processes, so metabolome changes during pregnancy may offer insights for reversing cachexia in cancer and even other metabolic disorders.

### Insulin resistance

Insulin, secreted by pancreatic β cells, is one of the most important hormones of metabolism, and its main function is regulating glucose metabolism. Insulin can increase the utilization of glucose and promote the synthesis of glycogen, adipose tissue and protein. Insulin resistance (IR) refers to a decrease in the efficiency of insulin to promote glucose uptake and utilization due to various reasons, including decreased insulin receptor phosphorylation when combined with insulin, decreased expression of insulin receptor substrate 1 (IRS-1), and decreased phosphoinositide 3-kinase (PI3K) response to insulin (135). IR is most common in diabetes but can also be observed in both pregnancy (136) and cancer cachexia (135, 137). IR tends to occur in the third trimester to maintain maternal euglycemia. During normal pregnancy, a nearly 50% reduction in insulin-mediated glucose disposal and increased insulin independence occurs with increasing gestational age (138). Changes in growth hormone (GH), human chorionic somatomammotropin (hCS) and several other blood sugar-regulating hormones lead to euglycemia during pregnancy. However, restoration of the balance between the decrease in hormones and increase in blood sugar may lead to chronic hyperglycemia or hypoglycemia (139). Even though the exact etiology of IR in pregnancy and cancer cachexia is not fully understood, some interesting similarities exist.

The development of IR in pregnancy is caused by imbalance of multiple hormones, including insulin-stimulating hormones, lactogenic hormones, prolactin and placental lactogens, which can promote the proliferation and secretion of pancreatic β cells, and hormones that induce IR, such as human placental prolactin lactogen (hPL), placental growth hormone (GH-2) (140) and insulin-like growth factor-1 (IGF-1) (141). In cancer cachexia, inflammation rather than tumor location, tumor attrition status, and tumor stage may play a key role in promoting IR (142). Chronic exposure to inflammatory cytokines, tumor necrosis factor α (TNF-α), IL-6, and IGFBPs can induce IR (143). The placenta and tumors, although employing different mechanisms, are sources of IR. After delivery of the placenta, maternal metabolism gradually returns to normal and similarly, if a tumor is treated or surgically removed, IR will gradually disappear (144, 145). However, the potential shared mechanisms between the placenta and tumors related to IR remain to be elucidated in further detail (146). IR is a mechanism related to cachexia (147). In tumors, IR occurs via suppression of the anabolic PI3K–AKT pathway and activation of the ubiquitin-mediated proteasome pathway (148), which in turn leads to muscle breakdown.

### Similarly altered circulating molecules

Exosomes are a newly identified method of cell-to-cell communication (149). One study innovatively identified some miRNAs carried by exosomes in maternal circulation that can be targeted to treat or prevent cancer. The study showed that exomiR-302d-3p, exomiR-223-3p and exomiR-451a, which are commonly associated with cancer metastasis and immune evasion, were highly expressed in pregnant women (150). Circulating cell-free DNA (cfDNA) contains short fragments of double-stranded DNA and is present in blood and other body fluids (151). Similar to miRNAs, cfDNA can also be packaged in exosomes (152) and can be released into the blood to maintain homeostasis and protect DNA molecules against nucleases and the immune system (153), and cfDNA can also reflect epigenetic changes in the body. There are similarities between cfDNA in cancer (154) and pregnancy (152, 155, 156). For example, hypermethylated RASSF1 can be found in the circulation in breast cancer, lung cancer and PE.

So far, studies have mainly focused on miRNA or cfDNA in exosomes. However, the impact of the placenta and tumor cells on the whole body reaches far beyond exosomes and miRNAs. Therefore, attention should also be paid to other kinds of molecules.

Metabolism during pregnancy is under precise regulation (157). Insufficient or excessive weight gain during pregnancy can lead to miscarriage, preterm gestational diabetes, and postpartum complications and can even affect offspring health (158). A study of the metabolomes of pregnant women at different gestational weeks demonstrated that metabolic reprogramming during pregnancy is related to many factors other than weight gain. The researchers found that 4,995 metabolic features (of 9,651 total), 460 annotated compounds (of 687 total), and 34 human metabolic pathways (of 48 total) were altered (157). This is the only study that reveals the characterization of the human pregnancy metabolome weekly and providing a high-resolution and detailed landscape. Another research used a serum and urine metabolomics approach to reveal the metabolic profile of cancer cachexia (159). More researches need to be done to reveal detailed landscape of pregnancy and cachexia. Another review suggested that metabolic abnormalities are shared by cancer and a variety of other chronic diseases that share underlying pathological processes. Cancer can share glucose-related, pyruvate-related and metabolism characteristics with diabetes, cardiovascular diseases, and neurological diseases (160). Metabolic abnormalities, including cachexia and hyperglycemia, can disrupt homeostasis, alter the body status, and in turn affect the development of tumors. Exploring the shared mechanisms between pregnancy and cancer cachexia by metabolomics may aid the development of new treatments.

One important molecule that is changed during pregnancy is steroid hormones, including the pregnancy-related hormones progesterone, estrogen, prolactin, and GH (161). It was once thought that these hormones mainly play roles in pregnancy maintenance, but it was later found that they were closely related to the occurrence and development of tumors, even several years after pregnancy, especially for tumors that develop in pregnancy-related organs, such as breast cancer (162), ovarian cancer and endometrial cancer (163). Carrying a pregnancy over 34 w can reduce the risk of breast cancer (162), which has been confirmed in a number of clinical studies.

## Conclusions

In this review, we attempted to summarize up the similarities between pregnancy and cancer in three levels. Embryogenesis and cancer metastasis share many cellular similarities (164). Trophoblast and cancer cells share gene mutations, EMT and invasion processes, cell–cell fusion features and other biological behaviors. The placenta can be thought of as a physiological tumor and is also considered a repository for damaged cells during embryonic development (6). More researches should be performed to explore the link between placenta and cancer. Regarding placenta as “pseudo-tumor” may be helpful to explore why accumulation of gene mutation in cancer becomes uncontrolled. Furthermore, the decidua and TME can both regulate epigenetics, migration, invasion, and immune tolerance and provide a hypoxic environment (12, 98). Uniquely, the placenta is removed from the decidua during childbirth or miscarriage. The decidua serves as a container that limits the overgrowth of the placenta while promoting its healthy development. Whether decidua, especially decidua of PE and RSA has antitumor effects is an interesting point to explore in future. Since decidua has powerful effect of regulating placenta development, if the differences or similarities of decidua and TME can be revealed in detail, it’s possible to reshape TME and find more ways to treat cancer can be found.

The last level of similarities between pregnancy and cancer is metabolites. Changes in metabolites in the circulation of cancer cachexia patients and pregnant women involve many of the same molecules, such as hormones and exosomes (150), but lead to totally different ending. IR is a feature shared by both patients with cachexia and pregnant individual (146, 147). If some metabolites of pregnancy have anti-cachexia effect, the future research can be done to identify some key molecules which can relieve cachexia and make survival with tumor realized. We hope our review can bring some new ideas to find cancer treatment targets.

## Author contributions

HP, DL, and YG wrote the draft. YY, TL, YL, and TC drew the figures. CF designed the study and helped draft the manuscript. All authors contributed to the article and approved the submitted version.

## Conflict of interest

The authors declare that the research was conducted in the absence of any commercial or financial relationships that could be construed as a potential conflict of interest.

## Publisher’s note

All claims expressed in this article are solely those of the authors and do not necessarily represent those of their affiliated organizations, or those of the publisher, the editors and the reviewers. Any product that may be evaluated in this article, or claim that may be made by its manufacturer, is not guaranteed or endorsed by the publisher.
